# Activation of Smooth Muscle K_ir_2.1 Channels and Na^+^/K^+^-ATPase Mediates Dilation of Porcine Coronary Arterioles at Physiological Levels of Potassium

**DOI:** 10.3390/ijms26062654

**Published:** 2025-03-15

**Authors:** Travis W. Hein, Habib M. Razavi, Xin Xu, Sonal Somvanshi, Mariappan Muthuchamy, Lih Kuo

**Affiliations:** Department of Medical Physiology, Cardiovascular Research Institute, College of Medicine, Texas A&M University Health Science Center, Bryan, TX 77807, USAmarim@tamu.edu (M.M.); lkuo@tamu.edu (L.K.)

**Keywords:** Na^+^/K^+^-ATPase, inward-rectifier potassium channels, resistance arterioles, potassium, vasodilation

## Abstract

Metabolic stress on the heart can cause dilation of coronary arterioles for blood flow recruitment. Although potassium ions (K^+^) released from the myocardium are a major mediator for this response, the underlying signaling pathways for vasodilation are incompletely understood. Herein, the roles of smooth muscle inward-rectifier K^+^ channel subtype 2.1 (K_ir_2.1) and Na^+^/K^+^-ATPase were examined. Porcine coronary arterioles were isolated, cannulated, and pressurized for vasomotor study. Vessels developed basal tone and dilated concentration-dependently to extraluminal K^+^ from 7 to 20 mM. Higher K^+^ concentrations (25–40 mM) caused graded vasoconstriction. Vasodilation to K^+^ (10 mM) was not altered by endothelial removal, and blockade of ATP-sensitive K^+^ channels, voltage-sensitive K^+^ channels, or calcium-activated K^+^ channels did not affect K^+^-induced vasodilation. However, sustained but not abrupt transient vasodilation to K^+^ was reduced by the nonspecific K_ir_ channel inhibitor Ba^2+^ or K_ir_2.1 channel blocker chloroethylclonidine. The Na^+^/K^+^-ATPase inhibitor ouabain attenuated K^+^-elicited vasodilation, and ouabain with Ba^2+^ abolished the response. Transfection of arterioles with K_ir_2.1 antisense oligonucleotides abolished sustained but not transient dilation. It is concluded that extraluminal K^+^ elevation within the physiological range induces initial transient dilation of porcine coronary arterioles by activating smooth muscle Na^+^/K^+^-ATPase and sustained dilation via smooth muscle K_ir_2.1 channels.

## 1. Introduction

Coronary blood flow is closely regulated to meet the metabolic demands of the heart by changes in the diameter of coronary arterioles. The coronary arteriolar diameter is influenced by myriad factors including the locally released metabolites from active cardiomyocytes. Potassium ions (K^+^) are one of the major metabolites released into the interstitial fluid and have been implicated in the regulation of vascular resistance in the coronary circulation [1,2,3]. During periods of acute myocardial ischemia [4,5,6,7,8] or increases in myocardial activity [2], interstitial K^+^ can increase from basal levels of 3–5 mM up to about 10–15 mM. The direct impact of K^+^ on vascular tone was supported by previous studies in isolated vessel preparations demonstrating that small modest increases in extraluminal K^+^ from 3 to 7 mM [9] or 14 mM [10] elicit dilation of pharmacologically preconstricted coronary arteries (260–700 µm). These in vitro studies demonstrated the capability of coronary conduit vessels in response to elevated K^+^. However, the sensitivity and reactivity of coronary resistance arterioles < 100 µm in resting diameter, the major site for coronary flow regulation, to small elevations in extracellular K^+^ within the physiological range (6 to 15 mM) has not been systematically investigated.

The underlying mechanisms implicated for K^+^-induced vasodilation include the activation of the Na^+^/K^+^-ATPase pump or the inward-rectifier K^+^ (K_ir_) channels. Early in situ studies [11] and isolated perfused heart [12] preparations indicate that intra-arterial infusion of isotonic KCl (at levels estimated to raise interstitial K^+^ concentrations by about 1–2 mM) causes coronary vasodilation, which is partially reduced by pharmacological blockade of Na^+^/K^+^-ATPase. By contrast, the activation of barium (Ba^2+^)-sensitive K_ir_ channels appears to be the predominant mechanism contributing to K^+^-induced vasodilation of isolated rat small coronary arteries (about 100–150 µm in resting diameter) [13]. However, it remains unclear whether activation of Na^+^/K^+^-ATPase and/or K_ir_ channels is attributable to the dilation of small coronary resistance arterioles to elevated K^+^. In addition, the relative cellular roles, i.e., the endothelium versus smooth muscle, in this vasodilation remain unknown. Vascular dilation in response to K^+^ was absent in mice lacking the gene encoding for K_ir_2.1 channels [14] and electrogenic Na^+^/K^+^ pump activity can alter cell membrane potential and influence vascular activity [15]. Therefore, we tested the hypothesis that elevation of K^+^ within the physiological range triggers the smooth muscle K_ir_2.1 channel subtype, in addition to activation of smooth muscle Na^+^/K^+^-ATPase, to elicit coronary arteriolar dilation. An isolated and pressurized microvessel preparation [16], coupled with videomicroscopic techniques [17,18], was utilized to eliminate the confounding influences from changes in hemodynamics and neurohumoral factors. The sensitivity and reactivity of porcine coronary arterioles to small increases in extracellular K^+^ and the cellular and molecular mechanisms underlying the vasodilation to K^+^ were characterized.

## 2. Results

### 2.1. Vasomotor Responses to Elevated Extraluminal K^+^

**Figure 1:**
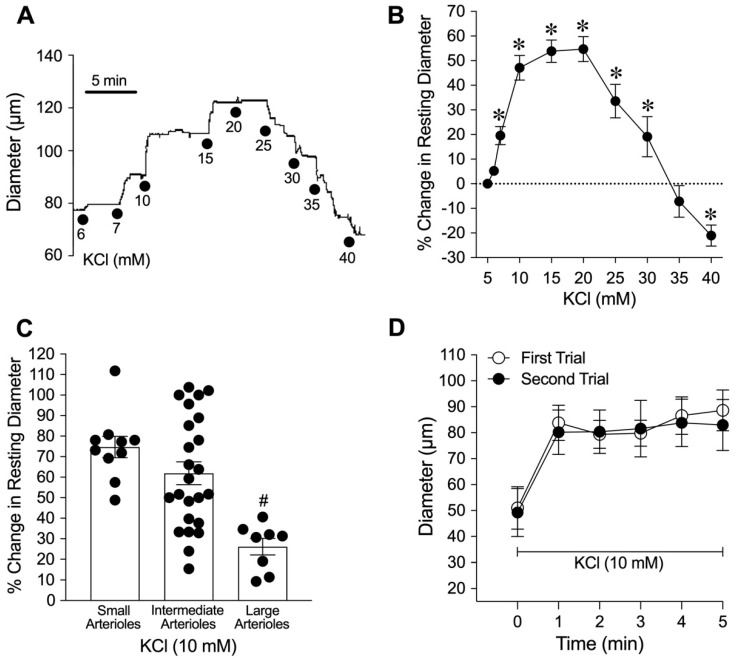
Vasomotor response of isolated coronary arterioles to elevations in extraluminal KCl. (**A**) Representative tracing shows the concentration-dependent response of an arteriole exposed to a stepwise increase in the concentration of KCl in the extraluminal bath from 5 to 40 mM. (**B**) Summary data show that extraluminal K^+^ concentrations of 6 (*n* = 13), 7 (*n* = 13), 10 (*n* = 19), 15 (*n* = 17), 20 (*n* = 15), 25 (*n* = 9), and 30 (*n* = 8) mM caused an increase in vessel resting diameter (vasodilation), whereas 35 mM K^+^ (*n* = 8) did not significantly alter resting diameter. Vasoconstriction occurred at 40 mM K^+^ (*n* = 9). * *p* < 0.05 versus the resting values (paired Student’s *t*-test). (**C**) Summary data of percent change in resting luminal diameter of small (34 ± 3 µm resting diameter, *n* = 10), intermediate (57 ± 2 resting diameter, *n* = 24), and large (98 ± 4 µm resting diameter, *n* = 8) coronary arterioles to 10 mM KCl. ^#^ *p* < 0.05 versus the small and intermediate arterioles (one-way ANOVA); *n*, number of vessels. (**D**) The changes in the diameter of small and intermediate coronary arterioles in response to 10 mM KCl were evaluated (First Trial) and then repeated after a 30 min washout period (Second Trial, *n* = 5). *p* > 0.05 between the First Trial and Second Trial (two-way ANOVA).

Within 60 min of equilibration, all isolated porcine coronary arterioles (*n* = 82) developed a similar level of basal tone (constricted to 60 ± 1% of maximum passive diameter 101 ± 3 μm; range 42–192 μm). Figure 1A shows the representative tracing of the arteriolar response to increases in the extraluminal concentrations of K^+^ from the baseline level of 5 mM up to 40 mM. Administration of KCl at various increments to the vessel bath (from 5 to 6, 7, 10, and 15 mM) caused a rapid cumulative increase in the vessel diameter (Figure 1A). No further increase in diameter was observed at 20 mM K^+^. Elevation of K^+^ to 25, 30, and 35 mM caused constriction of coronary arterioles back towards their resting diameter. At 40 mM K^+^, coronary arterioles constricted significantly below their resting diameter (Figure 1A). The changes in diameters were sustained at each concentration of K^+^ for 2 to 5 min in the observation period. The vasomotor reaction of coronary arterioles to K^+^ (ranging from 5 to 40 mM) is summarized in Figure 1B. In another series of experiments, to examine whether the vasodilation to K^+^ is vessel-size-dependent, the vessels were exposed to 10 mM KCl only. The dilation of small arterioles (resting diameter 17–43 µm) and intermediate arterioles (resting diameter 45–77 µm) to 10 mM KCl was comparable, but both responses were greater than those of large arterioles (resting diameter 81–110 µm) (Figure 1C). Time control experiments showed that vasodilation to 10 mM KCl was reproducible and did not deteriorate after repeated application (Figure 1D). Further studies were conducted below with 10 mM KCl to characterize the mechanism underlying the vasodilation.

### 2.2. Role of Endothelium

To explore the possible role of the endothelium in mediating the vasodilator response to K^+^, the vasomotor activity was assessed before and after the removal of the endothelium. The efficacy of endothelial disruption by 3-[(3-cholamidopropyl) dimethylammonio]-1-propanesulfonate (CHAPS, 0.4%) was verified by the absence of vasodilator response to 1 nM endothelium-dependent vasodilator bradykinin (control: 59 ± 5% dilation vs. CHAPS: 5 ± 2% dilation, *p* < 0.05, *n* = 5). Endothelial removal did not affect resting vascular diameter and vasodilations in response to elevated K^+^ (Table 1, Group 1).

### 2.3. Role of K^+^ Channels

**Figure 2:**
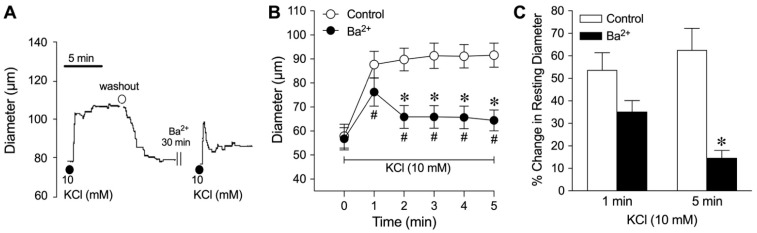
Role of K_ir_ channels in K^+^-induced dilation of isolated coronary arterioles. This data set was generated from arterioles with small, intermediate, and large luminal diameters. (**A**) Representative tracing shows the vasodilator response of an arteriole to 10 mM KCl before and after pharmacological blockade with the K_ir_ channel inhibitor Ba^2+^ (30 µM). (**B**) The changes in luminal diameter of coronary arterioles in response to 10 mM KCl were evaluated before (control) and after treatment with Ba^2+^ (30 µM, *n* = 9). * *p* < 0.05 vs. control response (two-way ANOVA); *^#^ p* < 0.05 versus the resting diameter at time point 0 (one-way ANOVA). (**C**) Summary data show the percent change in resting diameter at 1 min and 5 min after administration of 10 mM KCl in the absence (control) and the presence of Ba^2+^ (*n* = 9). * *p* < 0.05 vs. control response at 5 min (paired Student’s *t*-test).

The relative role of smooth muscle K^+^ channels in the coronary arteriolar dilation in response to K^+^ was assessed in the presence of their respective inhibitors. As shown in Figure 2A,B, administration of 10 mM KCl caused dilation of arterioles with a rapid increase in diameter reaching a plateau within 3 min that was sustained for at least 2 min. The arteriole regained tone after washing with PSS. Following treatment with Ba^2+^ (30 µM), administration of 10 mM KCl evoked an abrupt vasodilation within 1 min with the diameter returning towards the resting diameter and maintaining a small steady-state sustained dilation for at least 3 min (Figure 2B). These dynamic changes in diameters in response to 10 mM KCl are summarized in Figure 2C. Ba^2+^ did not alter the initial transient phase of K^+^-induced dilation within 1 min but significantly reduced the sustained dilation at 5 min (Figure 2). By contrast, the blockade of ATP-sensitive K^+^ (K_ATP_) channels with glibenclamide (5 µM, Table 1, Group 2), large-conductance calcium-activated K^+^ (K_Ca_) channels with iberiotoxin (0.1 µM, Table 1, Group 3), or voltage-dependent K^+^ (K_V_) channels with 4-aminopyridine (4-AP) (1 mM, Table 1, Group 4) did not influence the initial or steady-state components of the K^+^-induced vasodilator response.

### 2.4. Role of K_ir_2.1 Channels

**Figure 3:**
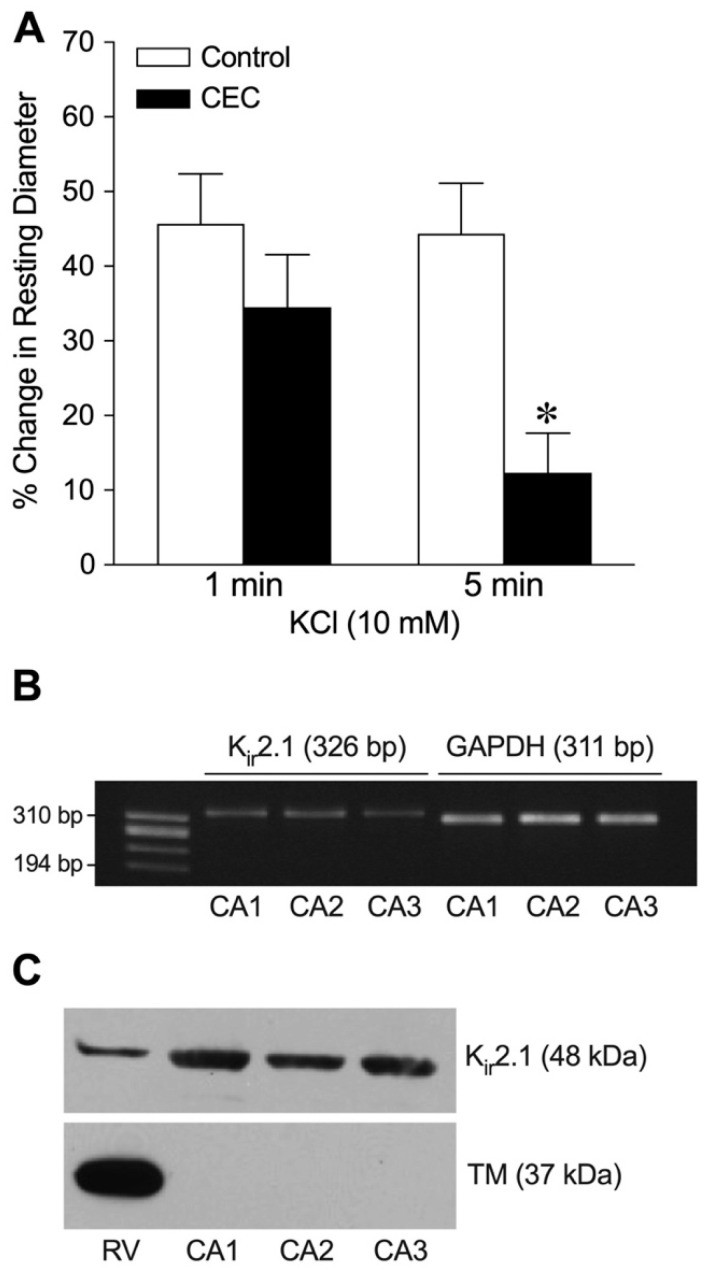
The role of K_ir_2.1 channels in K^+^-induced dilation of isolated coronary arterioles. This data set was generated from arterioles with small, intermediate, and large luminal diameters. (**A**) Summary data show the percent change in resting diameter at 1 min and 5 min after administration of 10 mM KCl in the absence (control) and presence of the pharmacological K_ir_2.1 inhibitor CEC (30 µM, *n* = 6). * *p* < 0.05 vs. control response at 5 min (paired Student’s *t*-test). (**B**) Total RNA in a single isolated coronary arteriole (CA, 60–80 µm in diameter in situ) from three different pigs (CA1, CA2, and CA3) was reverse-transcribed using gene-specific primers for K_ir_2.1 (326 bp) and GAPDH (311 bp) mRNAs. After PCR, gene products were electrophoresed on a 1.8% agarose gel and visualized with ethidium bromide staining. ϕX174 RF DNA/*Hae* III fragments were used as a size marker. (**C**) Western immunoblots were performed with proteins from right ventricle (RV) tissue and coronary arterioles (CAs) using anti-K_ir_2.1 and anti-tropomyosin (TM) antibodies.

Similar to the influence of Ba^2+^, treatment with the K_ir_2.1 channel inhibitor chloroethylclonidine (CEC, 30 µM) did not affect the extent of abrupt vasodilation at 1 min but inhibited the steady-state vasodilation at 5 min in response to 10 mM KCl (Figure 3A). The CEC blockade appeared to be specific because the inhibitor did not alter vasodilation to 1 µM pinacidil (control: 53 ± 5% dilation vs. CEC: 58 ± 10% dilation; *n* = 3) or 1 µM sodium nitroprusside (control: 52 ± 4% vs. CEC: 55 ± 4% dilation; *n* = 3). To support the functional studies above, the expression of K_ir_2.1 channels in porcine coronary arterioles was examined using reverse transcription–polymerase chain reaction (RT-PCR) and Western blot analyses. As shown in Figure 3B, K_ir_ 2.1 mRNA was detected in isolated coronary arterioles. Both K_ir_2.1 channel protein and striated/cardiac tropomyosin were detected in the right ventricular myocardium. In contrast, K_ir_2.1 channel protein, but not tropomyosin, was detected in coronary arterioles (Figure 3C).

**Figure 4:**
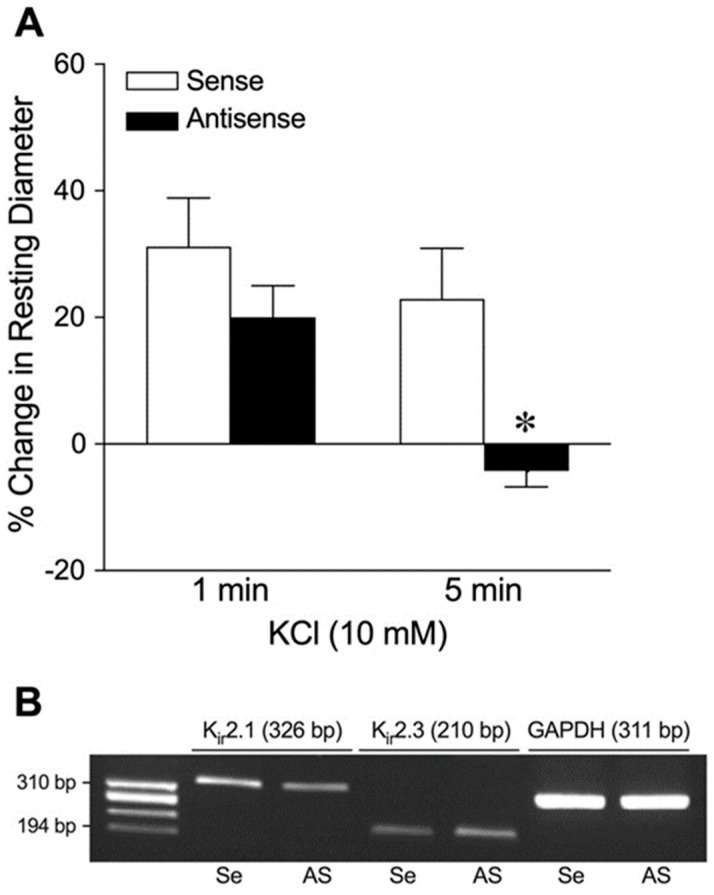
Assessment of K^+^-induced dilation of isolated coronary arterioles after K_ir_2.1 channel knockdown. This data set was generated from arterioles with small, intermediate, and large luminal diameters. (**A**) Summary data show the percent change in resting diameter at 1 min and 5 min after administration of 10 mM KCl in vessels transfected with sense (2.5 µg/mL, *n* = 5) or antisense (2.5 µg/mL, *n* = 5) oligonucleotides. * *p* < 0.05 vs. Sense response (paired Student’s *t*-test). (**B**) Total RNA from a single coronary arteriole (100 µm in diameter in situ) transfected with K_ir_2.1 sense (Se) or K_ir_2.1 antisense (AS) was reverse-transcribed using gene-specific primers for K_ir_2.1 (326 bp), K_ir_2.3 (210 bp), and GAPDH (311 bp) mRNAs. After PCR, gene products were electrophoresed on a 1.8% agarose gel and visualized with ethidium bromide staining. ϕX174 RF DNA/*Hae* III fragments were used as a size marker.

We determined the involvement of K_ir_2.1 channels in K^+^-induced vasodilation by corroborating the pharmacological studies with molecular knockdown of K_ir_2.1. Following a 24 h treatment of coronary arterioles with K_ir_2.1 sense oligonucleotides, the abrupt (1 min) and steady-state (5 min) dilations to 10 mM K^+^ were comparable (Figure 4A). By contrast, only an abrupt dilation was observed and sustained dilation was abolished in the vessels treated with K_ir_2.1 antisense oligonucleotides (Figure 4A). The vasodilator response to 1 µM sodium nitroprusside (sense: 54 ± 7% dilation, *n* = 3; antisense: 51 ± 15% dilation, *n* = 3) remained intact in the antisense-treated vessels. We also confirmed the fidelity of this molecular approach by specifically measuring K_ir_2.1 mRNA. As shown in Figure 4B, K_ir_2.1 and K_ir_2.3 mRNAs were detected in a single isolated coronary arteriole. Moreover, pretreatment with K_ir_2.1 antisense (As), but not K_ir_2.1 sense (Se), oligonucleotides reduced K_ir_2.1 transcripts without altering the expression of K_ir_2.3 and GAPDH (K_ir_2.1/GAPDH: sense, 0.39 ± 0.03 vs. antisense: 0.19 ± 0.08, *n* = 2).

### 2.5. Role of Na^+^/K^+^-ATPase

**Figure 5:**
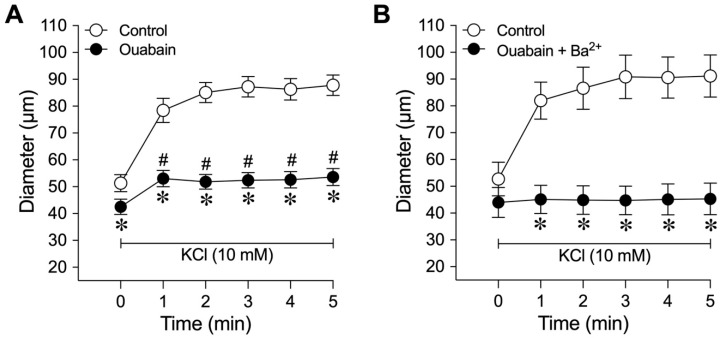
The roles of Na^+^/K^+^-ATPase and K_ir_ channels in the K^+^-induced dilation of isolated coronary arterioles. This data set was generated from arterioles with small and intermediate luminal diameters. The changes in diameter in response to 10 mM KCl were evaluated before (control) and after pharmacological blockade with (**A**) the Na^+^/K^+^-ATPase inhibitor ouabain (1.5 µM, *n* = 10) or (**B**) a combination of ouabain (1.5 µM) and the K_ir_ channel inhibitor Ba^2+^ (30 µM, *n* = 7). * *p* < 0.05 vs. control response (two-way ANOVA); *^#^ p* < 0.05 versus the resting diameter at time point 0 (one-way ANOVA).

The effect of the Na^+^/K^+^-ATPase inhibitor ouabain on K^+^-induced dilation was examined in a separate group of vessels. As shown in Figure 5A, pretreatment of the arterioles with ouabain (1.5 µM) significantly decreased the resting diameter (control: 51 ± 3 µm vs. ouabain: 43 ± 3 µm, *p* = 0.005, *n* = 10) and attenuated dilation to KCl. In the presence of ouabain, the abrupt and transient vasodilation was not seen, and the sustained vasodilation, with significantly reduced magnitude, remained (Figure 5A). It does not appear that ouabain elicited a nonspecific effect attributed to an increase in resting tone, because incubation of arterioles with 4-AP also significantly decreased resting diameter but did not alter K^+^-induced vasodilation (Table 1, Group 4). Pretreatment with both ouabain (1.5 µM) and Ba^2+^ (30 µM) abolished the arteriolar dilation to 10 mM KCl (Figure 5B).

## 3. Discussion

In the present study, five novel findings were noted regarding the vasomotor influence of K^+^ on isolated porcine coronary arterioles: (1) physiological concentrations of extracellular K^+^ in the range of 7 to 15 mM elicited a sustained, endothelium-independent coronary arteriolar dilation, with small and intermediate arterioles dilating to a greater extent than large arterioles; (2) both Ba^2+^ and CEC treatments inhibited the sustained vasodilation to K^+^, indicating a role of K_ir_ channels in the sustained vasodilator response to extraluminal K^+^; (3) molecular evidence of mRNA confirmed the presence of K_ir_2.1 in porcine coronary arterioles; (4) antisense knockdown of K_ir_2.1 channel mRNA abolished the sustained vasodilator response to K^+^, thus corroborating the pharmacological data; and (5) ouabain treatment blocked both the abrupt onset and sustained vasodilation to K^+^, suggesting that activation of the Na^+^/K^+^-ATPase pump plays a significant role in initiating the robust vasodilator response to K^+^.

In 1938, Katz and Linder reported that intra-arterial administration of K^+^ into the coronary artery of dogs produces coronary vasodilation, suggesting that coronary blood flow and metabolic demand may be closely related via K^+^ signaling in the heart [1]. Subsequent studies have shown that the byproducts of tissue metabolism, including extracellular K^+^, dilate blood vessels to match perfusion to metabolism in several tissues, including the heart [19,20]. Experimental studies in animals have demonstrated that coronary arterial occlusion triggers the dilation of small coronary arterioles, and this dilation causes blood flow recruitment immediately after the release of the occlusion. Thus, coronary blood flow is abruptly increased to compensate for the oxygen debt during occlusion. This phenomenon of reactive hyperemia is thought to be mediated via several metabolites. It is well recognized that a wide array of factors, such as carbon dioxide, acidosis, adenosine, decreased oxygen tension, and increased interstitial osmolarity, in addition to K^+^, can contribute to coronary blood flow regulation by altering coronary arteriolar diameter, especially in response to metabolic stress [21,22,23,24,25]. Although the cellular/molecular mechanisms of the above putative metabolic vasodilators in evoking coronary arteriolar dilation have been investigated previously [18,21,22,23,24,25,26,27,28], the mechanistic action of K^+^ on those microvessels remains incompletely understood. Interestingly, the extracellular concentration of K^+^ in the heart rises immediately after the onset of acute coronary artery occlusion and can reach 10–15 mM within 6 to 15 min in the region of myocardial ischemia [4,5,6,7,8]. While the above in vivo studies support a potential role of K^+^ in coronary flow regulation, the responsiveness and mechanism of coronary arteriolar dilation to elevated K^+^ remain unclear. Notably, the in vivo preparations are associated with systemic/local hemodynamic and/or neurohumoral changes, as well as allow the microvessel segments to have direct longitudinal interactions within the network and horizontal interactions with the surrounding cardiomyocytes during experimental interventions. These introduced factors can influence coronary vasomotor activity and/or reactivity [25,29,30,31,32,33,34] and complicate data interpretation regarding the characterization of responsiveness and the underlying mechanisms involved. The present study used an isolated vessel approach to directly address the responsiveness and cellular/molecular mechanism of coronary arterioles subjected to K^+^ challenge. Our results demonstrate that extracellular K^+^ concentration in the 10–15 mM range causes abrupt coronary arteriolar dilation and could possibly contribute to this local reactive hyperemic response. Likewise, increases in extracellular K^+^ may trigger functional hyperemia during increased heart rate. An earlier study has shown that myocardial pacing in the dog caused a nearly 1–2 mM increase in the coronary interstitial level of K^+^ that preceded the increase in coronary blood flow [2]. The authors suggested that this small elevation in the K^+^ concentration might initiate rather than maintain the functional hyperemic response. However, they were also uncertain whether a 1–2 mM increase in the local level of K^+^ is sufficient to evoke the dilation of coronary arterioles. Our present findings support this possibility and provide the first direct evidence that an increase in the extraluminal concentration of K^+^ from the normal level of 5 to 7 mM can elicit a nearly 20% increase in the resting diameter of porcine coronary arterioles. Because the flow is proportional to the 4th power of vessel radius changes, a 20% increase in caliber can elicit a 2-fold increase in blood flow. Taken together with the previous in vivo studies, it appears that small elevations in the extracellular concentration of K^+^ in the physiological range of 7 to 15 mM can have a significant impact on local coronary vascular resistance, and thus blood flow in vivo.

The endothelium is well known to mediate and/or modulate smooth muscle activity, especially in the microvessels because the ratio of endothelial to smooth muscle cells is markedly increased in the microvascular network and the vascular cells are intimately exposed to the tissue metabolites. Endothelial denudation significantly reduced the relaxation of isolated small mesenteric arteries (100–200 µm) in response to 7.8 to 13.8 mM K^+^ [35], suggesting a role for the endothelium in this K^+^-induced response. However, it is unclear whether these observations can be applied to the regulation of vasomotor activity by K^+^ in the coronary arterioles. In the isolated coronary arteriolar preparation, we found that the endothelium is unlikely to be the target of the K^+^ for vasodilation, because the K^+^-elicited response was not altered by endothelial removal, a result consistent with other studies in the small coronary and cerebral arteries [13,36]. Our results in coronary arterioles provide further evidence that the K^+^-induced dilation, at least in the coronary microcirculation, originates from the smooth muscle cell.

In the present study, the dilation of coronary arterioles to K^+^ was sensitive to a low concentration of nonspecific K_ir_ channel inhibitor Ba^2+^ and the K_ir_2.1 channel blocker CEC (Figure 2 and Figure 3). Indeed, several other studies in the cerebral [13,36], skeletal muscle [37], cremasteric [38], and small coronary [13] arteries have shown that Ba^2+^ inhibits the K^+^-induced vasodilator response. In the present study, as well as other previous studies, Ba^2+^-sensitive K^+^-induced dilation was resistant to inhibitors of K_ATP_, K_Ca_ and K_V_ channels, suggesting that K^+^ was acting through the K_ir_ channel [13,39]. In support of an unequivocal role for the K_ir_ channels in the regulation of coronary microvascular blood flow, the density of the K_ir_ current has been shown to be inversely related to blood vessel size [40,41,42]. Our findings show that small and intermediate arterioles about 30–60 µm in resting diameter exhibit greater dilation in response to 10 mM K^+^ than their upstream parent vessels (>100 µm in resting diameter) (Figure 1C), supporting the concept of the dominant role of small resistance arterioles in metabolic regulation of coronary blood flow [25,29]. Although the pharmacologic data pointed to the important role of K_ir_ in mediating K^+^-induced coronary arteriolar dilation, the limitation of selectivity and specificity of pharmacologic blockers must be considered. Importantly, our functional data were also corroborated with molecular evidence at the level of mRNA expression, ascertaining that K_ir_2.1 channel subtypes are not only present in the coronary arterioles but also mediate the vasodilation in response to K^+^. This was specifically demonstrated by the knockdown of K_ir_2.1 channel mRNA using antisense oligonucleotides, which correspondingly matches the reduction of K^+^-induced vasodilation. Although it was not studied in the coronary microvasculature, the importance of K_ir_ channels was shown with K_ir_2.1 gene disruption in mice, resulting in the loss of inwardly rectifying K^+^ currents and K^+^-induced dilation in murine cerebral arteries [14]. Without K_ir_2.1, the coronary arterioles constricted to a low concentration of KCl (10 mM) (Figure 4A). It is worth noting that K_ir_2.1 deficiency may contribute, in part, to the development of hypertension [43] and possibly the impairment of coronary [44] and cerebral [45] flow regulation under increased metabolic activity of the tissue. It appears that K_ir_2.1 channels contribute significantly to the vasomotor influence by K^+^ in the organs that predominantly rely on metabolic flow regulation.

In our study, inhibition of K_ir_2.1 channels revealed two components of vasodilation elicited by K^+^, i.e., an abrupt/transient phase of initial dilation (e.g., ~1 min after the addition of KCl) and a sustained/prolonged phase of dilation (e.g., 2–5 min after the addition of KCl). For example, antisense K_ir_2.1 channel knockdown, as well as pretreatment of the coronary arterioles with Ba^2+^ or CEC, allowed for an abrupt transient dilation but reduced the sustained component (Figure 2, Figure 3 and Figure 4). Therefore, K_ir_ channel blockade did not abolish the initial dilation response, pointing to another putative mechanism that contributed to the activation of K_ir_ channels in K^+^-induced vasodilation. We found that ouabain alone abolished the abrupt transient dilation to K^+^ and almost abolished the sustained dilation (Figure 5A). The vasodilator response to K^+^ was completely abolished when ouabain was combined with Ba^2+^ (Figure 5B). These data, along with our molecular findings, support the idea that a small elevation of K^+^ activates Na^+^/K^+^-ATPase for abrupt and transient vasodilation and subsequently triggers the opening of K_ir_2.1 channels for sustained dilation. We previously showed that sodium azide evokes coronary arteriolar dilation via the activation of K_ir_ channels and Na^+^/K^+^-ATPase [46]. This vasodilation can be inhibited by morin, an inhibitor of phosphatidylinositol phosphate (PIP) kinase [47]. Since K_ir_ channel activity can be regulated by PIP kinase [48,49,50] and the K^+^-induced dilation in isolated coronary arterioles is attenuated by morin in a manner similar to Ba^2+^ [46], it is likely that PIP kinase links to Na^+^/K^+^-ATPase and K_ir_ signaling for coronary arteriolar dilation to elevated K^+^.

It should be noted that ouabain slightly but significantly caused constriction of coronary arterioles under resting conditions, indicating a tonic inhibition of Na^+^/K^+^-ATPase in the development of basal vascular tone in these vessels. However, the increased vascular tone did not appear to affect vasomotor activity, because administrating the Kv channel inhibitor 4-AP significantly increased vascular tone to a level similar to that of ouabain, but the K^+^-induced vasodilation (10 mM) was no different than untreated controls. In small cerebral arteries, K^+^ concentrations from 0 to 5 mM caused activation of Na^+^/K^+^-ATPase pumps, hyperpolarization, and dilation [36]. The transient nature of the response was attributed to K^+^ saturation of the Na^+^/K^+^-ATPase pump, Na^+^ extrusion and hyperpolarization, followed by reaching a new steady state [13,36,51,52]. Because there are at least four isoforms of Na^+^/K^+^-ATPase with varying sensitivities to external K^+^ [53], the specific molecular role of each Na^+^/K^+^-ATPase isoform in mediating coronary arteriolar dilation in response to K^+^ remains unclear. For instance, a previous study has shown expression of Na^+^/K^+^-ATPase α_1_, α_2_, and α_3_ isoforms in rat mesenteric artery myocytes, where only α_2_ and α_3_ subtypes are responsible for hyperpolarization due to increments in external K^+^ concentrations [53]. In our study, the identity of the Na^+^/K^+^-ATPase isoform(s) that may account for the initiation of vasodilation to K^+^ remains to be probed.

In addition to the limitation of selectivity and specificity of pharmacologic blockers, other factors need to be considered for data interpretation in the present study. For example, the RT-PCR technique used for analyses of K_ir_ channels is semi-quantitative due to challenges associated with the linearity of band density measurements and signal saturation. The extremely small amount of total RNA harvested from a single arteriole for the molecular study of K_ir_ channels (*n* = 2) also posed a challenge in performing rigorous statistical analyses. Furthermore, our study did not stratify data based on the age (juvenile pigs used in this study) and sex of the animals, so we cannot preclude that some of the variance noted in K^+^-evoked segmental responsiveness is sex/age-dependent. It is unclear whether the observed vessel responses are also applicable to the coronary circulation of adult or aged animals. The link of PIP kinase to the Na^+^/K^+^-ATPase isoform and the dynamic opening of K_ir_ channels for vasodilation in response to K^+^ also deserves further investigation.

In summary, the present study shows that elevated extraluminal K^+^ concentrations, within the physiological levels, are associated with marked dilation of coronary arterioles inversely related to vessel size. The increased vascular responsiveness to K^+^ with decreased arteriolar size highlights the importance of the longitudinal increase in vasomotor activity for coronary blood flow regulation in response to metabolic stress. The vasodilator response to K^+^ is independent of other K^+^ channels and the endothelium. The induction of initial transient dilation of porcine coronary arterioles by activating smooth muscle Na^+^/K^+^-ATPase appears to trigger sustained vasodilation via smooth muscle K_ir_2.1 channels. Although the precise molecular signaling between Na^+^/K^+^-ATPase activity and K_ir_ remains unclear, the activation of PIP kinase appears to be involved. These vasodilator mechanisms are expected to play important roles in response to the elevated K^+^ during metabolic activation or stress, including ischemia and hypoxia.

## 4. Materials and Methods

### 4.1. Animals and Chemicals

All animal procedures adhered to the approved guidelines set by the Texas A&M University and Baylor Scott & White Health Institutional Animal Care and Use Committees (ID: 2003-029-R and 2007-033-R). Pigs (8–12 weeks old of either sex; 10–20 kg; *n* = 66) purchased from Barfield Farms (Rogers, TX, USA) or Oak Hill Genetics (Ewing, IL, USA) were sedated with Telazol (4–8 mg/kg, intramuscularly; TW Medical Veterinary Supply, Austin, TX, USA) and then anesthetized and anticoagulated with an intravenous administration of pentobarbital (30 mg/kg) and heparin (1000 U/kg; Cardinal Health, Dublin, OH, USA), respectively. Following a thoracotomy, the heart was excised and immediately placed in cold (5 °C) saline. Drugs were obtained from Sigma (St. Louis, MO, USA) unless otherwise stated. Rauwolscine and CEC were dissolved in water. Glibenclamide was dissolved in dimethyl sulfoxide, whereas pinacidil, ouabain, and 4-AP were dissolved in absolute ethanol. Subsequent concentrations of these drugs and all other drugs were dissolved in PSS. The final concentrations of dimethyl sulfoxide and ethanol in the tissue bath were 0.03% and 0.1%, respectively. Vehicle control studies indicated that these final concentrations of solvent had no effect on arteriolar function.

### 4.2. Isolation and Cannulation of Coronary Microvessels

Because coronary arterioles are sensitive to changes in local hemodynamics [16,17,30] and neurohumoral factors [25], the individual arterioles were studied in vitro in the present study to eliminate these confounding influences that are inevitable within in vivo preparations. Subepicardial arteriolar branches of the left anterior descending artery (about 1 mm in length; 30 to 100 µm in internal diameter in situ) were dissected from the surrounding right ventricular myocardium and were cannulated with glass micropipettes as previously described [29,54]. The vessels were pressurized to 60 cmH_2_O intraluminal pressure, consistent with the in vivo level of coronary arteriolar pressure [31,55], and bathed in physiological salt solution (PSS) (mM: NaCl 145.0, KCl 5.0, CaCl_2_ 2.0, MgCl_2_ 1.17, NaH_2_PO_4_ 1.2, glucose 5.0, pyruvate 2.0, EDTA 0.02, and MOPS 3.0) containing 1% albumin (USB Corporation, Cleveland, OH, USA) as described previously [30]. The inner diameter of coronary arterioles was measured using video microscopic techniques and recorded with a PowerLab data acquisition system (ADInstruments, Colorado Springs, CO, USA) [16,30].

### 4.3. Pharmacological Assessment of K^+^-Induced Vasodilation

Cannulated coronary arterioles were bathed in PSS–albumin at 36–37 °C to allow the development of basal tone. The vessels constricted to about 50–70% of maximum diameter within a 60 min equilibration period and maintained a stable resting diameter. After a stable basal tone was developed, the concentration-dependent responses to incremental increases in the extraluminal K^+^ concentration (5 to 40 mM) were assessed. The extraluminal K^+^ concentration (5 mM in PSS) was increased by adding KCl to the vessel chamber with final concentrations of 6, 7, 10, 15, 20, 25, 30, 35, and 40 mM in the vessel bath. The osmolality of the solution was maintained constant by equimolar decreases in the NaCl concentration (5 mM K^+^: 300 ± 1.4 mOsmol vs. 20 mM K^+^: 300 ± 1.0 mOsmol) [32]. Further studies on the evaluation of signaling pathways were performed at 10 mM because this concentration of K^+^ is consistent with that found in the interstitium of the heart under metabolic stress [4,5,6]. To confirm the reproducibility of the vasodilation to 10 mM KCl, the response of some vessels was re-examined after 30 min. In another cohort, the K^+^-induced dilation was examined after endothelial removal by perfusion of a nonionic detergent, CHAPS (0.4%), as previously described [54]. Only vessels that exhibited normal basal tone, did not dilate in response to the endothelium-dependent agonist bradykinin (1 nM), and showed unaltered vasodilation in response to the endothelium-independent agonist sodium nitroprusside (1 nM to 100 µM) after endothelial removal were accepted for data analysis.

In other vessels, the contributions of K^+^ channels and Na^+^/K^+^-ATPase to the K^+^-induced dilation were evaluated in a series of experiments by administration of their cognate inhibitors to the extraluminal bath for at least 30 min. The roles of K_V_ channels, K_ATP_ channels, and K_Ca_ channels were assessed in the presence of their respective inhibitors 4-AP (1 mM) [46,56], glibenclamide (5 µM) [18], and iberiotoxin (0.1 µM) [54]. The role of K_ir_ channels in K^+^-induced dilation was assessed in the presence of 30 µM BaCl_2_ (a specific inhibitory concentration for K_ir_ channels) [13,32,40]. Furthermore, the contribution of K_ir_2.1 channels was assessed in the presence of its inhibitor CEC (30 µM) [39,57]. Since CEC can also activate α_2_-adrenoceptors, which can cause the dilation of coronary arterioles [58], vessels were incubated with both CEC and an α_2_-adrenoceptor antagonist rauwolscine (5 µM) [59]. Our preliminary studies showed that rauwolscine alone prevented vasodilation in response to CEC but did not alter vasodilation in response to 10 mM KCl (control: 45 ± 3% change in resting diameter vs. rauwolscine: 49 ± 10% change in resting diameter, *n* = 2). The role of the Na^+^/K^+^-ATPase in the vasodilator response to K^+^ was determined in the presence of its specific inhibitor ouabain (1.5 µM) [46]. To examine the specificity of pharmacological inhibitors in the K^+^-induced effect, vasodilations in response to sodium nitroprusside and the K_ATP_ channel opener pinacidil in the presence of these inhibitors were studied in some vessels.

### 4.4. Antisense Knockdown of K_ir_2.1 Channels

To directly identify a functional role for K_ir_2.1 channels, the coronary arteriolar dilation in response to K^+^ was examined in the presence of antisense or sense oligonucleotides according to the Superfect Transfection Reagent protocol (Qiagen, Germantown, MD, USA) [60,61]. Coronary arterioles (60–100 µm in diameter in situ) were carefully dissected and incubated in Dulbecco’s Modified Eagles Medium containing 10% fetal bovine serum, 1% antibiotic–antimycotic (Sigma), and Superfect Transfection Reagent (20 µL/mL) for 3 h at 37 °C in the absence and presence of antisense or sense oligonucleotides (2.5 µg/mL) for K_ir_2.1 channels (22 base pairs corresponding to bases 381–402 of gene accession no. AF153820, synthesized by Sigma-Genosys) [62]. After this incubation period with the transfection–oligonucleotide complexes, the media was removed, and the vessels were then incubated with the media alone for 24 h. The vessels were subsequently cannulated and pressurized for functional assessment in response to K^+^. RT-PCR analysis was performed as described below to verify the efficacy of K_ir_2.1 blockade by antisense oligonucleotides.

### 4.5. RNA Isolation and RT-PCR

Total RNA was isolated from individual porcine coronary arterioles (60–100 μm in diameter in situ, 2–3 mm in length) as previously described [26,63]. Briefly, the vessel was homogenized in 1 mL Trizol Reagent (Life Technologies, Rockville, MO, USA), and total RNA was isolated according to the manufacturer’s instructions with slight modification. The total RNA sample (400–500 µL) isolated from Trizol/chloroform extraction was then coprecipitated with 5 µL of glycogen (25 µg/mL; Invitrogen, #AM9510) to optimize total RNA recovery during the overnight precipitation step with isopropyl alcohol plus 50 µL sodium acetate (5 M) [64]. Sets of primers specific for K_ir_2.1 (gene accession no. AF153820, sense: 5′-TGA CAA CGC AGA CTT TGA AAT CGT-3′; antisense: 5′-TCT GGA ACT CCA TTT TCA CTG TCG-3′), K_ir_2.3 (gene accession no. NM0044981, sense: 5′-ACG AGA ACG AGC TGG CCC TTA TGA-3′; antisense: 5′-ACT CCC TGC GGT AGG AGA TGT TGT-3′), and GAPDH [26,63] were used. Specifically, 0.3 and 0.1 µg of total RNA for K_ir_ and GAPDH samples, respectively, were annealed to the 3′-specific primers, and the RT reaction was performed using Thermoscript reverse transcriptase (Life Technologies). Five microliters of RT cDNA were subsequently used to perform PCR for 30 (GAPDH) or 35 (K_ir_ channels) cycles with Expand High Fidelity PCR enzyme (Roche, Indianapolis, IN, USA). Electrophoresis of PCR-amplified products on a 1.8% agarose gel followed by treatment with ethidium bromide allowed for band visualization. Images of stained products were acquired with the Gel Doc 2000 system (Bio-Rad Laboratories, Hercules, CA, USA). The level of K_ir_ channel transcripts was normalized to that of GAPDH transcripts.

### 4.6. Western Blot Analysis

Isolated coronary arterioles (5 vessels pooled, 40–100 μm in diameter in situ, 1–3 mm in length) and right ventricular myocardial tissue were sonicated in lysis buffer containing 50 mM Tris-HCl, pH 7.5, 0.1 mM EDTA, 0.1 mM EGTA, 1 μg/mL leupeptin, 1 μg/mL aprotinin, and 0.1 mM phenylmethylsulfonyl fluoride as previously described [26]. The protein content of each tissue lysate was determined using a BCA protein assay kit (Pierce, Rockford, IL, USA). Briefly, 4 micrograms of protein per lane were separated by 10% SDS-PAGE under reducing conditions. After electrophoresis, proteins were transferred onto a nitrocellulose membrane (Bio-Rad Laboratories), followed by blocking overnight at 4 °C with 5% dry milk in PBS. Immunodetection was then achieved by allowing the membranes to react with primary antibodies against K_ir_2.1 (rabbit anti-K_ir_2.1, 1:250, Alomone Labs, catalog # APC-026, Jerusalem, Israel) or striated (cardiac/skeletal muscle) tropomyosin (mouse anti-tropomyosin, 1:1000, Abcam, catalog # ab7786) overnight at 4 °C. The anti-tropomyosin antibody reacts with cardiac α-tropomyosin but not smooth muscle isoforms of tropomyosin, so it was used to test for contamination of microvessel samples with cardiomyocytes. After washing with TBS for 45 min, the secondary antibody labeled with horseradish peroxidase was administered for 1 h at room temperature. The blots were revealed with horseradish peroxidase-labeled goat anti-rabbit (1:1000) or anti-mouse (1:2000) IgG secondary antibodies (Jackson ImmunoResearch Laboratories, West Grove, PA, USA) by an enhanced chemiluminescence assay (Amersham Pharmacia, Piscataway, NJ, USA).

### 4.7. Data Analysis

At the end of each functional experiment, the vessel was dilated with sodium nitroprusside (100 µM) in an ethylenediaminetetraacetic acid (1 mM)-containing calcium-free PSS after the washout of pharmacological inhibitors to obtain its maximum diameter at 60 cmH_2_O intraluminal pressure [65]. For the analysis of vasomotor responses, internal diameter changes were normalized to the resting basal diameter and expressed as a percent change in resting diameter. Data are reported as mean ± SEM, and *n* values represent the number of vessels studied (one vessel per pig for each treatment). Statistical comparisons of vasomotor responses and resting vascular tone before and after various treatments were performed using a paired Student’s *t*-test or one-way or two-way analysis of variance (ANOVA) with the Dunnett or Bonferroni multiple-range test as deemed appropriate. A value of *p* < 0.05 was considered significant.

## Figures and Tables

**Table 1:** Effect of endothelial denudation and K^+^ channel blockers on the dilation of porcine coronary arterioles to an increase in extraluminal KCl.

Intervention	*n*	Resting Diameter (µm) 5 mM KCl	% Change in Resting Diameter 10 mM KCl
*Group 1*	5		
Control Denudation		57 ± 6 57 ± 7	48 ± 8 43 ± 6
*Group 2*	5		
Control Glibenclamide		63 ± 9 60 ± 10	38 ± 6 47 ± 13
*Group 3*	3		
Control Iberiotoxin		72 ± 12 70 ± 13	38 ± 5 41 ± 8
*Group 4*	4		
Control 4-AP		60 ± 9 45 ± 5 *	33 ± 7 55 ± 14

Values are mean ± SEM; *n* = number of vessels. Diameter changes were normalized to resting diameter at 5 mM KCl and expressed as % change in resting diameter after 5 min exposure to 10 mM KCl. Denudation (0.4% CHAPS); glibenclamide (5 µM); iberiotoxin (0.1 µM); 4-AP (1 mM). * *p* < 0.05 vs. control.

## Data Availability

The data sets generated and/or analyzed for the current study are available from the corresponding authors upon reasonable request.
